# Investigating the Association between Unhealthy Dietary Habits and Obesity among Libyan Adults

**DOI:** 10.3390/ijerph19031076

**Published:** 2022-01-19

**Authors:** Hamdi Lemamsha, Gurch Randhawa, Chris Papadopoulos

**Affiliations:** 1 Faculty of Medical Sciences, University of Omar Al-Mukhtar, Al-Bayda Campus, Labraq Road, Al-Bayda B1L12, Libya; dr_hamdi_lemamsha@outlook.com; 2 Institute for Health Research, University of Bedfordshire, Putteridge Bury Campus, Hitchin Road, Luton LU2 8LE, UK; chris.papadopoulos@beds.ac.uk

**Keywords:** obesity, BMI, Libya, eating habits, eating behavior, food portion sizes, skipping breakfast

## Abstract

Background: Although an increasing number of studies have reported on nutrition transition and unhealthy eating habits (UEHs) worldwide, there is a paucity of studies on UEHs in the Arab region, particularly in Libya. *Aim:* This study investigated the associations between obesity among Libyan adults and UEHs. *Methods:* A cross-sectional survey was conducted at the five major districts in Benghazi, Libya. A multistage cluster sampling strategy was implemented to choose and recruit Libyan adults. Anthropometric measurements were gathered by highly qualified nurses, using the Segmental Body Composition Monitor and a portable Stadiometer. The study used and adapted the two Self-administered questionnaires: the WHO STEPS Instrument and eating behaviors linked with obesity questionnaire. *Results*: Among a total of 401 participants who were successfully recruited in this study, 253 (63%) were female (aged 20–65 years), the response rate achieved was 78%. The prevalence of obesity amongst Libyan adults was estimated to be 42.4%. The results revealed the presence of a significant association between obesity (BMI ≥ 30 kg/m^2^) and five UEHs for Libyan men and six UEHs for Libyan women. For Libyan men, an association was found between obesity and the following five explanatory factors: fast food intake in a day and a week, which were (OR: 2.52, 95% CI: 4.04–12.32) and (OR: 4.65, 95% CI: 1.04–9.46), respectively; large food portion sizes consumed at one sitting (OR: 19.54, 95% CI: 1.41–27.74); a high frequency of skipping breakfast either in a typical day or a week, which were (OR: 0.02, 95% CI: 0.01–0.77) and (OR: 0.03, 95% CI: 0.01–0.24), respectively. For Libyan women, a significant association was found between obesity and the following six explanatory factors: fast food intake in a day and a week, which were (OR: 2.14, 95% CI: 3.32–11.12) and (OR: 5.5, 95% CI: 1.88–16.11), respectively; intake of sugar-sweetened beverages in a typical week (OR: 4.02, 95% CI: 1.35–11.99); and large food portion sizes consumed at one sitting at one sitting (OR: 3.40, 95% CI: 1.18–9.84); and a high frequency of skipping breakfast either in a typical day or a week, which were (OR: 0.11, 95% CI: 0.03–0.43) and (OR: 0.12, 95% CI: 0.08–0.63), respectively. *Conclusions*: The findings of the study reveal areas of action for Libyan researchers, clinicians, policymakers, and government officials about UEHs in the Libyan context. This could inform establishing and developing new interventions for preventing and controlling the obesity epidemic through food system improvements.

## 1. Background

Obesity is a complex, multifaceted global public health issue. It affects individuals of all ages, gender, races, and nations and is the fifth biggest cause of death worldwide [1,2]. According to the World Health Organisation (WHO) (2021), global obesity prevalence has more than doubled in 70 nations since 1980 and approximately over 650 million adults globally are obese (for 13% of the world’s adult population). Likewise, the prevalence of obesity worldwide has nearly tripled since the mid-1960s, accordingly, the WHO predicted that by 2025, one in every five adults globally is anticipated to be obese [2,3]. In Libya, obesity has emerged as a critical health issue due to the significant risk of severe co-morbidities, potentially leading to premature mortality. Additionally, as well as health consequences, obesity has a number of psychological, social, and economic consequences [4,5,6].

Libya is considered to have the highest Human Development Indices (HDI) in Africa and its HDI is ranked 55th in the world, this has caused Libya to be listed with high-income countries in relation to different development measures, for instance, life expectancy, education, and income per capita [4,5]. Since oil was discovered in Libya late in the 1950s, the country has followed the trend of transition in the economic, demographic, and epidemiological spheres. These transitions have a collective impact on the health status of the Libyan population, contributing to this nutrition transition that includes a poor diet and sedentary lifestyle [4,7]. The nutrition transition can be characterized by cheap calories, animal foods, refined grains, sugary drinks, and fast-food restaurants [8,9]. Consisting of a high intake of red meat, refined sugars, and saturated fat with little fiber, the impact of this nutrition transition is spreading worldwide [7,8].

The nutrition transition is considered an important factor that can affect dietary intake through unhealthy eating habits (UEHs), particularly in many middle-income countries such as Libya. This can result in a rising epidemic of overweight and obesity among adults and adolescents, as well as widespread diet-related, non-communicable diseases [7,8]. While studies in developed and industrial countries have demonstrated extensively how UEHs tend to promote obesity [7,8,10], there is a dearth of evidence in developing countries including Libya. Therefore, addressing this topic in the present study could help to elucidate the relationship between UEHs and obesity among the Libyan population in particular, and in the Middle East and North Africa (MENA) population in general.

The prevalence of obesity (BMI ≥ 30 kg/m^2^) in Libya has more than doubled in the last three decades, from 12.6% in 1984 to 30.5% in 2014 [11]. Accordingly, obesity has become a public health issue in the Libyan context because obesity causes a major risk for fatal diet-related diseases leading to disability, morbidity, and mortality in the Libyan adult population. Furthermore, obesity has adverse social, psychological, and economic implications, which lead to increased economic losses. As a result, obesity has imposed a substantial economic burden on the Libyan state as a whole, which was estimated to be 65% of the Libyan state health budget [4,5,11]. When considering factors that may contribute to or protect against obesity, it is important to consider both sides of the energy-balance equation: on the one hand, the factors related to energy intake (involving food consumption and beverage intake) and, on the other hand, the factors related to energy expenditure (involving physical activity and sedentary lifestyle) [12,13]. It has been suggested that the more risk factors an individual has, the greater the chance of that person developing obesity and dying from it [14]. It is therefore vital that we increase our understanding of the risk factors (for example, UEHs) that might contribute to obesity, particularly in an under-researched population such as Libyan adults, who possess different cultural norms from those typically studied.

Although healthy foods (high nutrient-dense foods) are generally more expensive than unhealthy foods (less nutrient-dense food options) [15,16], in the Libyan context, the scenario can be somewhat different from neighboring countries in that food is sometimes available, affordable, and accessible to all Libyans, meaning that high nutrient-dense foods are potentially no longer difficult to obtain [17]. However, this situation is not necessarily stable, considering the current political situation between rival militias which can result in a rapid deterioration of living conditions including restricted access to high nutrient-dense foods [18,19].

Due to the strong existing evidence on the association between obesity and UEHs (skipping breakfast and the consumption of large food portion sizes at one sitting, etc.), the hypothesis was derived, made, and applied to the Libyan context. Therefore, we have hypothesized that there is a significant association between obesity (BMI ≥ 30 kg/m^2^) in Libyan adults and five main UEHs, operationalized as excessive consumption of fast foods; excessive consumption of sugar-sweetened beverages (SSBs); eating less than five daily portions of fruit and vegetables; a high frequency of skipping breakfast; and consumption of large food portion sizes (FPS) at one sitting. Therefore, this study aimed to investigate the associations between obesity among Libyan adults and the five aforementioned unhealthy eating habits.

## 2. Methods

### 2.1. Study Design and Setting

The study design was a cross-sectional survey was undertaken in the second-largest city, Benghazi, at five parliamentary constituencies (Al-Keisha; Al-Sabre; Al-Salmani-ElSharki; Bu Atni; and Madinat Benghazi). In comparison to other Libyan cities, Benghazi is a prosperous city with a high level of urbanization and modernization. Being the economic and trade capital of Libya, Benghazi is ethnically diverse and multicultural [4,11].

### 2.2. Participants and Procedure

Inclusion criteria for the study were as follows: being aged between 20 and 65 years with a good level of Arabic language skills; resident in Benghazi for several years with their names on the electoral register (roll) in Benghazi to vote in an election or referendum; pregnant women, potential participants who are unstable on their feet such as chair-bound, amputees, as well as those who have an injury to the legs, feet, spine, or brain; and those who are too fragile or unable to stand straight were excluded.

The sampling procedure comprised of three stages: sampling from parliamentary constituencies, polling districts, and ultimately the electoral register. To comply with the Libyan High National Election Commissions’ (HNEC) (2014) regulations, the 11 parliamentary constituencies of Benghazi were utilized as clusters. Al-Break, Al-Keisha, Al-Sabre, Al-Salami-El-Garb, Al-Salmani-El-Sharki, Al-Uruba, Benghazi al-Jadida, Bu Alni, Benina, Garyounis, and Madinat Benghazi are the 11 parliamentary constituencies listed in alphabetical order from (A–Z). Primary Sampling Units (PSUs) were acquired in the first step by a systematic random selection of five of the eleven constituencies named alphabetically and allocated serial numbers. Al-Keisha; Al-Sabre; Al-Salmani-El-Sharki; Bu Atni; and Madinat Benghazi were the five constituencies. Each parliamentary constituency is divided into three polling districts in accordance with Benghazi Municipal Election regulations (2012). Secondary Sampling Units (SSUs) were generated in the second stage through a simple random sampling of one polling district from each of the five constituencies (PSUs), namely, Al-Fuwayhat (population: 8012); Al-Kwayfiya (population: 10433); Raas Abayda (population: 8480); Laithi (population: 11858); and Al-Hadaa’iq (population: 19102). The total population of the five selected polling districts was 57885. Finally, in the third stage, final or Tertiary Sampling Units (TSUs) were determined by systematic random sampling of possible participants.

The sample size of this study was computed using the following formula [20]: Sample Size (SS) = $\frac{1.96^{2}\left(0.5\right)\times \left(1-0.5\right)}{0.05^{2}}$ = 384 Libyan adults. In order to enhance the achieved response rate, for this study, it was assumed that a predictable response rate (RR) for this study could be 75%. Since the minimum sample size of the study was computed and estimated to be 384 people and the likely response rate proposed was 75%, thus, the final estimated sample size for this study was 384/0.75 = 512.

**Figure 1:**
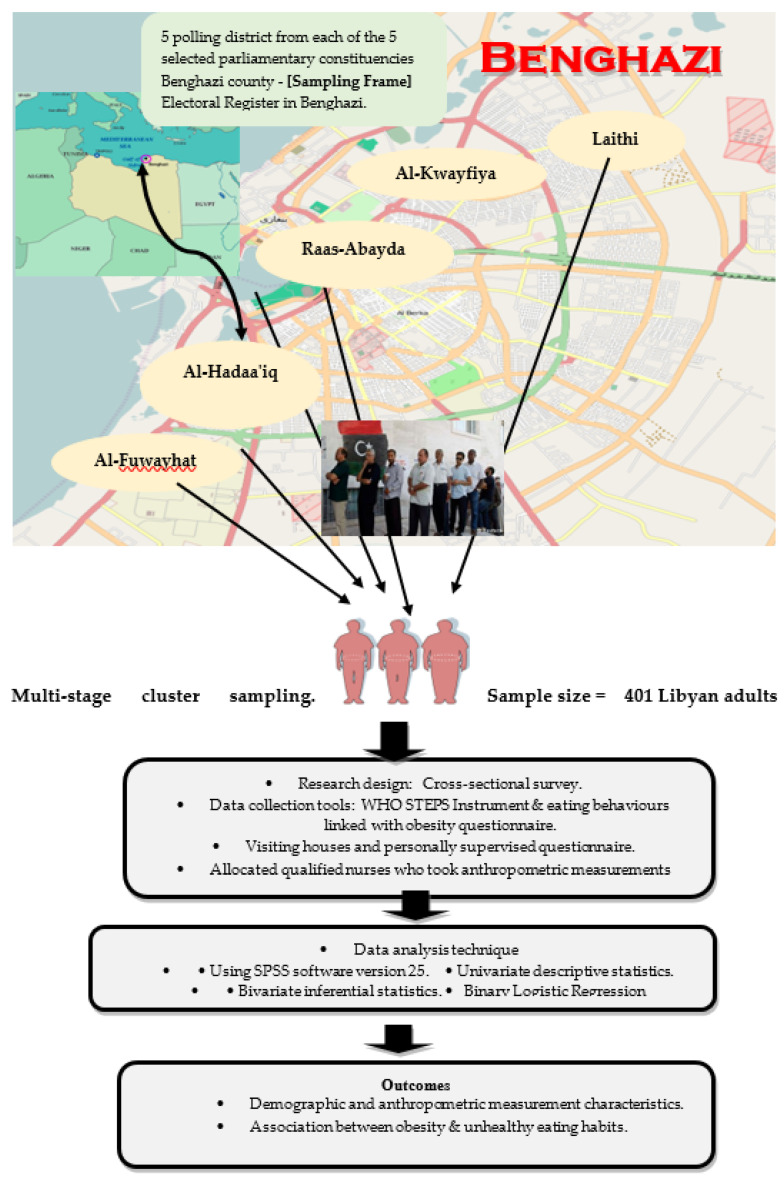
A graphical scheme of study design.

Two approaches were implemented in order to inform all participants who were selected from the electoral register about the opportunity to participate or refuse to participate in the forthcoming survey. The two approaches utilized: (1) a pre-notification (introductory) letter was delivered to 165 (32%) 110 participants through a reliable communication agency that belonged to the Libyan general postal service; and (2) the majority 347 (68%) participants were informed by phone. The questionnaires were distributed to the participants by hand (and participants were given the opportunity to accept or decline participation) under the researcher’s supervision during the field visit. In order to enhance the reliability of obesity measurements gathered for Libyan adults (from 15 August 2014 to 20 December 2014), the researchers assigned ten highly qualified nurses (two for each district) who took anthropometric measurements from the participants and recorded these measurements in the assigned section of the questionnaire [21]. See Figure 1.

### 2.3. Ethical Considerations

Three Ethical approvals letters were granted from Research Ethics Committees that were affiliated to the three Institutions: Institution for Health Research at the University of Bedfordshire, UK [Ref. No: IHREC303/2014], Omar Al-Mukhtar University, Bayda, Libya, [Ref. No: UOA-44/2014], and the Regional Health Ministry in Benghazi, Libya [RHMb-24/2014]. Each participant received written information regarding the nature and purpose of the study. Prior to the investigation commencing, informed consent was obtained in writing.

### 2.4. Measures

#### 2.4.1. Anthropometric Measuring Tools

A portable stadiometer was used in this survey, to measure the height (cm) to the closest 0.1 cm. Weight to the nearest 0.1 kg, BMI (kg/m^2^). Additionally, Percent Body Fat to the nearest 0.1 percent and Visceral Fat Level were all measured using the Tanita BC-601 Segmental Body Composition Monitor.

#### 2.4.2. Sociodemographic Characteristics

The WHO STEPS Instrument for Noncommunicable Disease Risk Factor Surveillance [22], the Arabic translated version (scale of Information on sociodemographic and socioeconomic status (SES) variables) was used to evaluate participants’ sociodemographic (age, gender, ethnic origin, and religion) and SES (measured by three factors: education level, income, and occupational status).

#### 2.4.3. Unhealthy Eating Behavior Assessment

This survey adopted and used Greenwood et al.’s [10] questionnaire for eating behaviors associated with overweight and obesity. This questionnaire has been tested for validity and reliability and used as an effective screening tool in order to examine the association of these UEHs with obesity. Our adapted unhealthy eating behavior questionnaire contains five items: (1) fast food (Foods with a high energy density) consumption; (2) consumption of sugar-sweetened beverages; (3) consumption of fruits and vegetables; (4) skipping breakfast; (5) consumption of large food portion sizes (FPS) at one sitting.

### 2.5. Translation Technique

This study implemented a forward-backwards translation technique [23] in order to translate all documents related to this survey (pre-notification letter, the participant information sheet (PIS), the informed consent form (ICF), and two adapted questionnaires) from English into the Arabic language by allocating three Libyan certified translators (LT1, LT2, and LT3). Additionally, this technique entails appointing an expert committee (consisting of one health professional, an Arabic proofreader, as well as all three translators) in order to evaluate the backwards-forward translation technique and ensure that the final Arabic edited version is reliable, valid, and ready to use.

### 2.6. Statistical Analysis

Numeric data analysis was performed using the Statistical Package for Social Sciences (SPSS, version 25.0; SPSS Inc.: Chicago, IL, USA). In this study, the univariate analysis, bi-variate inferential statistical tests, and a binary logistic regression were conducted to analyze the quantitative data. Univariate analysis and Descriptive statistics analyses were conducted to describe the response of participants’ socio-demographic data, socioeconomic status (SES), as well as participants’ UEHs. Secondly, Spearman’s rank-order correlation was implemented to investigate the correlation used between obesity and the eight out of nine variables of UEHs, while Chi-square (X^2^) was performed to investigate the association between obesity and large FPS intake. Thirdly, logistic regression analysis was performed to examine the relationship between UEHs and obesity in Libyan adults and analyzed according to the gender variance after adjusting for socio-demographic factors and SES. Additionally, a binary logistic regression analysis was performed to identify which, if any, UEHs independently predicted obesity. Odds ratio (OR) with 95% confidence interval (CI) was computed to assess the presence and degree of association between obesity (BMI ≥ 30 kg/m^2^) and UEHs. The statistical significance level was set at 5%. Goodness-of-fit statistics are employed to determine whether the model adequately describes the data. The Hosmer–Lemeshow statistic indicates that the model poor fit the data if *p* < 0.05.

The sociodemographic and socio-economic and UEHs variables were converted into binary items in order to perform a binary logistic regression analysis, which was performed to examine the relationship between the outcome variable (BMI) and UEHs, see Table 1 for details. BMI was coded into two categories as follows: not obese (BMI < 30 kg/m^2^) and overweight and obese (BMI ≥ 30 kg/m^2^), and each of the following predictor variables was coded: sociodemographic and SES components (education level, income, or occupational status) [24,25]; and unhealthy eating behavior [26,27].

### 2.7. Validity and Reliability of the Two Questionnaires

Content validity and face validity were implemented by allocating an expert committee (two translators, two public health experts, two healthcare professionals) to evaluate the two translated adopted questionnaires after the backwards-forward translation. The expert committee concurred that the final edited Arabic version of the obesity questionnaire for Libyan adults possessed the following characteristics: straightforward; understandable; all the questions are in a logical sequence; easy for the respondents to answer; meaningful. While to test construct validity, Pearson’s correlation was employed. The two questionnaires: the WHO STEPS Instrument and the UEHs questionnaire were tested for construct validity in a pilot study, and a significant correlation was found between the score for each item of the questionnaire and the total score of the same questionnaire for each questionnaire was as follows: the WHO STEPS Instrument (r = 0.521, *p* < 0.001); the UEHs questionnaire (r = 0.279, *p* < 0.001). The reliability (internal consistency) was tested for the two instruments using Cronbach’s alpha. Generally, the coefficient should be >0.7 to be considered relatively high in most social science research situations [28]. The internal consistency reliability of the WHO STEPS instrument (scale of sociodemographic and SES) was relatively high (Cronbach’s α = 0.90). The reliability of the UEHs instrument was also relatively high (α = 0.92).

## 3. Results

This section presents the finds in the form of descriptive Demographic, SES, and anthropometric measurement characteristics along with UEHs among Libyan adults. Secondly, it presents the results of testing hypotheses by using correlation and association between obesity and UEHs. Finally, it describes the binary logistic regression models which show the impacts of UEHs on obesity. The study successfully recruited 401 Libyan adults from the five districts as follows (Al-Keisha = 51; Al-Sabre = 75; Al-Salmani-ElSharki = 54; Bu Atni = 86; and Madinat Benghazi = 135). The actual response rate achieved in the study was 78%, which was slightly higher than the estimated response rate (75%).

Table 2 summarizes the demographic characteristics and anthropometric measurement of the 401 participants aged 20–65 years old. A total of 401 Libyan adults participated and completed the questionnaire, with a response rate of 78%. The majority were female (63.1%, *n* = 253), married (67.1%, *n* = 316), and Arab (84.6% *n* = 339). The highest age group of those surveyed was 40 to 49 years. More than half of those surveyed (51.4%) were with higher levels of educational attainment; and with 77.6%, the majority worked in Governmental or non-governmental institutions. Over half of the adults (51%) reported that they earned a high income. The mean BMI of those surveyed was 29.52 (±6.19) kg/m^2^, the mean visceral fat was 10.42 (±4.1), and the mean Body Fat Percentage was 31.57 (±9.42%). See Table 2 for a full breakdown of this data. Finally, Table 2 also shows that the prevalence of obesity amongst Libyan adults was 42.4%, whereas the prevalence of obesity was 33.8% and 47.4% among Libyan men and women, respectively.

Table 3 illustrates the means of UEHs in a typical day/week of those surveyed. The average frequency of fast food intake among Libyan adults was 1.37 (±0.8) times for a typical day and 5.54 (±3.1) times for a typical week. The mean of sugar-sweetened beverages (SSBs) intake among Libyan adults was 3.15 (±1.2) cans for a typical day and 11.68 (±1.23) cans for a typical week. The average number of portions of fruit and vegetables consumed by Libyan adults was 2.53 (±1.2) portions in a typical day and 10.1 (±1.6) portions for a typical week. The average frequency of breakfast skipped among Libyan adults was 0.5 (±0.3) on a typical day and 3.18 (±1.83) in a typical week. One hundred and sixteen (41.4%) of Libyan adults reported that they are always consuming large food portion sizes served in one sitting, this sample included 95 (37.5%) Libyan women and 71 (48%) Libyan men. The table also shows that a significant positive correlation was found between obesity and four UEHs, namely: fast food intake (rho = 0.440, *p* = 0.000); intake of SSBs ‘in a typical day (rho = 0.467, *p* = 0.000); times breakfast intake (rho = −0.506, *p* = 0.000); consumption of large FPS at one sitting (X^2^ = 13.8, *p* = 0.000). In contrast, a lack of association was found between obesity and amount of vegetable and fruit intake (rho = 0.081, *p* = 0.107).

## 4. Summary and Interpretation of Binary Logistic Regression Model

Model Summary presents the variation in BMI (outcome variable) explained by the UEHs (independent variables) with Cox and Snell R^2^ and Nagelkerke R^2^ (Pseudo R^2^) values. The Cox and Snell R^2^ = 0.65 shows 65% variation in BMI is explained by the predictors but the remaining 0.35% is unexplained. These R^2^ values demonstrate the explained variation in BMI, by a set of UEHs, ranging from 65% (Cox and Snell R^2^) to 90% (Nagelkerke R^2^) for males and ranging from 42% (Cox and Snell R^2^) to 68% (Nagelkerke R^2^) for females. Additionally, it demonstrates the −2 Log-Likelihood statistic as 30.09 for males and 101.19 for females. Additionally, the goodness-of-fit of the model for both genders is non-significant (X^2^ = 0.55, df = 8, *p* = 1.00) for male and (X^2^ = 4.67, df = 8, *p* = 0.79) for female. The results show the non-significant X^2^ value *p* > 0.05, thus, it can be concluded that the model is good fit of data.

Table 4 shows the impact of UEHs on obesity among Libyan adults of both genders using binary logistic regression. After adjusting for socio-demographic and SES factors, statistical associations were found between obesity and five of the nine UEHs. A significant association was found between obesity and the following UEHs: fast food intake in a typical day (OR: 3.14, 95% CI: 1.04–9.46; *p* = 0.04); fast food intake in a typical week (OR: 6.83, 95% CI: 2.59–12.01; *p* < 0.001). While negative association was found between obesity and a high frequency of skipping breakfast in a typical day (OR: 67, 95% CI: 0.03–0.25; *p* < 0.001 *p* = 0.03); and a high frequency of skipping breakfast in a typical week (OR: 0.08, 95% CI: 0.45–0.67; *p* < 0.001). Additionally, a significant association was found between obesity and large FPS consumed at one sitting (OR: 4.78, 95% CI: 84–12.46; *p* < 0.001). In contrast, a lack of association was found between obesity and the remaining UEHs: SSBs intake either in a typical day or week and vegetable and fruit intake either in a typical day or week. Notably, fast food intake in a week was the strongest predictor of outcomes compared to other factors in this model linked to increased obesity.

Table 5 summarizes the association between UEHs and BMI using binary logistic regression across gender-related variances when sociodemographic and SES factors were adjusted. The results revealed the presence of a significant association between obesity and five UEHs for Libyan men and six UEHs for Libyan women. For Libyan men, a positive association was found between BMI and the following three explanatory factors: fast food intake in a day (OR: 2.52, 95% CI: 4.04–12.32; *p* = 0.047) fast food intake in a week (OR: 4.65, 95% CI: 1.04–9.46; *p* = 0.014); large FPS consumed at one sitting (OR: 19.54, 95% CI: 1.41–27.74; *p* = 0.027). In contrast, a negative relationship was found between BMI and a high frequency of skipping breakfast either in a typical day or a week for men, which were (OR: 0.02, 95% CI: 0.01–0.77; *p* = 0.042) and (OR: 0.03, 95% CI: 0.01–0.24; *p* = 0.011), respectively.

For Libyan women, a positive association was found between BMI and the following four explanatory factors: fast food intake in a day(OR: 2.14, 95% CI: 3.32–11.12; *p* = 0.043); fast food intake in a week (OR: 5.5, 95% CI: 1.88–16.11; *p* < 0.001); intake of sugar-sweetened beverages (SSBs) in a typical week (OR: 4.02, 95% CI: 1.35–11.99; *p* = 0.013); and large FPS consumed at one sitting (OR: 3.40, 95% CI: 1.18–9.84; *p* = 0.024). In contrast, a negative relationship was found between BMI and a high frequency of skipping breakfast either in a typical day or a week for females, which were (OR: 0.11, 95% CI: 0.03–0.43; *p* < 0.001) and (OR: 0.12, 95% CI: 0.08–0.63; *p* < 0.001), respectively. Notably, large FPS consumed at one sitting in males was the strongest predictor of outcomes compared to other factors in this model, linked to increased risk of obesity.

## 5. Discussion

This section discusses and interprets the association between obesity (BMI ≥ 30 kg/m^2^) among the Libyan adults population and each of the five unhealthy eating habits, operationalized as follows: excessive consumption of fast foods; excessive consumption of SSBs; eating less than five daily portions of fruit and vegetables; a high frequency of skipping breakfast; consumption of large food portion sizes (FPS) at one sitting. This cross-sectional study successfully recruited 401 Libyan adults from the five districts in Benghazi. The actual response rate achieved was 78%. This study found that the prevalence of obesity amongst Libyan adults was 42.4% (for a prevalence of obesity at 47.4% in women compared to 33.8% in men).

### 5.1. Fast-Food Consumption

This study establishes a strong, significant positive association between obesity and the frequency of consuming fast food, whether in a typical day or in a week, in the adult Libyan population of both genders. One possible explanation for the positive association between obesity and the frequency of consuming restaurant and fast food is that most fast foods have an extremely high energy density, and are palatable (less nutrient-dense foods options become more desirable), energy-dense foods are associated with reduced satiation and satiety [29,30]. Being affordable, convenient, and accessible, fast food encourages a large number of Libyans to purchase it as an alternative to expensive, healthier food [4,5]. Moreover, the contribution of factors like emotional eating among other eating disorder subgroups have been shown in a previous study to have an existing correlation with BMI [31]. In studies conducted to identify motivations of food choices across different countries and potential cross-cultural interactions, sensory appeal and price were the most important motivations reported of food choices, while the least important were ethical concern familiarity, mood and natural content [32,33]. Another possible factor that contributes to the high rate of fast-food consumption is the availability of relatively homogeneous fast food outlets across Benghazi [4,5,9]. In addition, some fast-food restaurants advertise more aggressively and extensively than others, which may encourage vast numbers of Libyans to eat out at these particular restaurants [4,6,9]. Our findings are aligned with those of various epidemiological studies conducted in developed countries [34,35,36], Arab countries [37,38,39], which found that frequent fast-food consumption was positively associated with increased BMI.

### 5.2. Consumption of Sugar-Sweetened Beverages (SSBs)

The study establishes a strong, significant positive association between obesity and the frequency of consuming SSBs in a typical day, in Libyan adults of both genders. In contrast, after adjusting for all probable confounding factors, consumption of SSBs was significantly associated with obesity in Libyan women but not in men. One possible explanation for the excessive consumption of SSBs amongst Libyans is the fact that SSBs are inexpensive, palatable, energy-dense foods [40,41]. Apart from being readily available and heavily promoted through media advertising, sugary drinks provide energy but do not seem to induce satiety [42]. Another explanation for high levels of SSBs consumption among Libyan adults is that the inclination for less nutrient-dense foods increases in social situations, which are key triggers for food and beverage consumption [43]. Traditional Arab hospitality dictates that a variety of SSBs are served as refreshments to guests on social occasions. A further explanation is that, during the summer months, the hot and arid climate in Libya leads to dehydration which affects Libyans’ health. Consequently, Libyans seek out cold SSBs from grocery stores and vending machines so as to rehydrate [4,42]. In addition, SSBs are considered to be a staple commodity and a priority for all Libyan families; hence, they are offered at each meal [4,6].

The results were unexpected in terms of finding no association between the consumption of SSBs and an increase in BMI in men, despite the average consumption of SSBs for a Libyan adult being 3.68 (±1.23) cans a day The findings demonstrated that the average consumption of SSBs by Libyan adults was 545 kcal of SSBs per person per day—the equivalent of 3.6 cans of SSB per day (1.278 mls or 43.2 ounces). This amount exceeds the recommended daily amount of SSBs by the American Heart Association, which is no more than 450 kilocalories (kcal) of SSBs: the equivalent of approximately three 12-ounce cans per week [44]. Likewise, the WHO recommends that the consumption of SSBs should be limited to less than a single can of SSBs per person per day [45].

Our findings were consistent with numerous cross-sectional studies in both developed and developing countries, which revealed a positive association between the consumption of SSBs and obesity in women but not in men [46]. However, the most common pattern in the literature is the finding of no gender differences in obesity rates related to the consumption of SSBs. For example, a large prospective cohort study among adults conducted in developed societies found that higher SSB consumption was associated with significant weight gain for both genders [47]. Furthermore, other epidemiological studies conducted in developed and developing countries, found that SSB intake among adults is significantly associated with greater adiposity [48,49].

### 5.3. Eating Less than Five Daily Portions of Fruit and Vegetables

According to the WHO [50], high consumption of fruit and vegetables (FV) per day (defined as at least five portions) is associated with a reduced risk for obesity. However, this study found no association between consuming five daily portions of fruit and vegetables and obesity in the adult Libyan population of either gender. Numerous explanations can be posited for the lack of association revealed in this study between a high daily intake of FV and a reduced risk of obesity in Libyan adults. One possible explanation states that those five portions are insufficient to have an effect; recently obesity experts and public health activists have advised that doubling the FV intake of five- or seven-a-day to ten-a-day would be a more optimum intake level [51,52]. Hence, it may be argued that more than five portions a day are required for a higher protective effect against obesity [51]. It is somewhat surprising that the average daily FV intake among Libyan adults is 3.13(±1.42) portions a day, which is relative low—particularly with respect to the new recommended level proposed by the NHS [51] and considering that fruit and vegetables are accessible, affordable and available to low-income minority families and wealthy communities alike in Libya [53].

Another possible explanation is that, although Libyans reported a slightly acceptable level of daily FV intake, they tended not to reach the recommended minimum advised by the Public Health England, the NHS [51]. This may be because Libyan eating habits entail the consumption of FV after heavy meals and after they have reached the satiety stage, after which they may take a nap [54,55]. All these unhealthy eating behaviors may influence metabolism and the absorption process so that their bodies cannot utilize the fruit and vegetables that they do consume; hence, the way in which they consume fruit and vegetables may be contributing to the increase in weight among Libyans rather than reducing weight [52,56]. Therefore, the BMI of Libyan adults is not associated with the consumption of five daily portions of FV, in either gender.

The findings of the study indicate that the average FV intake among Libyan adults was 3.13(±1.42) portions a day, which is slightly lower than the WHO recommendation of at least 400g of fruit and vegetables per day, equating to approximately five portions, where one portion weighs about 80 g [50,51]. Despite this manageable level of FV intake, the findings revealed no association between daily FV intake and obesity. The average reported daily FV intake among Libyan adults in the study was lower than that in another EMR country, namely, Iran where the mean daily FV intake is 4.58 ± 1.31 and 4.65 ± 1.28, for men and women, respectively [57]. Our results align with those of Charlton et al. [58], Moradi-Lakeh et al. [59] which found no significant association between fruit and/or vegetable intake and obesity. However, the findings are unexpected because they are contrary to a substantial body of literature including previous epidemiological studies—both cross-sectional and prospective human cohort studies—which found a significant inverse correlation between intake of five daily FV portions and the risk of obesity [60,61,62]. In contrast, several studies have established a significant positive correlation between daily FV intake and obesity [63,64,65].

### 5.4. A High Frequency of Skipping Breakfast

The study found a significant negative association between breakfast consumption per week and obesity in the adult Libyan population of both genders. Furthermore, after adjusting for all probable confounding factors, the same inverse association between breakfast consumption per week and obesity was found in both genders. There are two possible explanations for an inverse association between breakfast consumption per week and the lower BMI in Libyan adults in both genders. First, it has been suggested that eating breakfast helps to reduce the tendency towards unplanned, impulsive snacking and hence there is less consumption of the calories and fats associated with spontaneous snacking [66,67,68]. Thus, skipping breakfast is a significant factor in re-setting the body’s biological clock and generally results in people feeling nourished and satisfied. In sum, those who eat breakfast are less likely to overeat throughout the rest of the day [64,65,66]. Second, skipping breakfast may affect people’s physiological functioning in that fasting results in stress and disruption to the metabolic process, leading people to attempt to compensate later in the day through excessive consumption of foods high in either saturated fats or refined carbohydrates, which may contribute to an increase of obesity [64,65,69]. This finding aligns with the literature in that several epidemiological studies conducted in developed and developing countries, including Arab countries, revealed compelling evidence for an inverse association between a high frequency of skipping breakfast and obesity [70,71,72].

### 5.5. The Consumption of Large Food Portion Sizes

This study reveals a strong significant positive association between the consumption of large FPS at one sitting and obesity in the adult Libyan population of both genders. One explanation for a positive association between the consumption of large FPS at one sitting and obesity in Libya is that large FPS provide an excess of calories for the body in a single sitting, which can contribute to individuals’ putting on weight [4,73]. The higher the frequency of consuming large FPS at one sitting, the higher the BMI in Libyan adults, was an unsurprising result due to the general lack of health awareness in Libya [4,5], which may result in increasing ignorance about what constitutes an appropriate FPS at one sitting. Another explanation for consuming large FPS at one sitting is that in addition to under-estimating the calories in large FPS at one sitting, many people adhere to the expectation to “clean our plate”, leading eventually to obesity [4,74]. According to the etiquette of eating in Islam, people are taught to serve food in small portions and must not leave even a bit of their food for ‘Satan’ [75,76]. The plate of food should be finished without leftovers because nobody knows in which portion of the food the blessing from God lies [73]. However, most Libyans misunderstand the etiquette concerning the consumption of large FPS at one sitting. In keeping with Libyan culture, the food served must be highly palatable and in large portions, as this is how the host demonstrates their hospitality to guests [4,52]. Consequently, Libyan etiquette forces people to consume all food that is served [4,52]. The excess energy intake from this practice arguably contributes to the development of obesity. Our findings are consistent with the findings of epidemiological studies, which concluded that the consumption of large FPS at one sitting was positively associated with overweight and obesity in adults [77,78].

## 6. Limitations

The first limitation is that the cross-sectional methodology precluded drawing explicit directed causal conclusions since it was snapshot research, which may result in contradicting findings if a different period was used to repeat the survey [27]. Additionally, self-reported data and the nature of cross-sectional data may introduce recollection and reporting biases, which may account for the lack of significance in obesity-related causes [79]. Arguably, the broad eligibility criteria for the research participants included in this study adversely affected the response rate in that several groups were excluded due to either physical health problems or practical reasons, for example, advanced techniques were required for measuring the BMI of certain groups such as pregnant women; amputees; and, if their BMI was obtained, it may vary from the standard BMI cut-point values. Additionally, a portable Tanita BC-601 Segmental Body Composition Monitor cannot provide BMI readings for the following groups: pregnant women, amputees, and those who are unable to stand straight or unsteady on their feet [4]. Furthermore, the study’s exclusion of the aforementioned categories for practical reasons is probably another limitation. Finally, the study was undertaken pre-coronavirus disease (COVID-19) pandemic and as a result, a major limitation is an uncertainty of how these findings now apply by the Libyan population in the context of the current COVID-19 outbreak.

The research contributes to our understanding of unhealthy eating behaviors that lead to weight gain not only in Libya but also in other Arab countries which share similar food trends, cultural norms, and religious practices. Furthermore, this study also identified risk factors associated with obesity that can be utilized by Libyan health policymakers in developing prevention and intervention programs to reduce the obesity epidemic in the Libyan adult population.

## 7. Conclusions

The findings of this study confirmed that obesity is one of the fastest-growing and most serious public health challenges confronting the Libyan government. The study found that the Libyan population are at risk of consuming unhealthy diets (fast food intake; skipping breakfast; and consuming large portions), which are likely to contribute to the obesity epidemic in both genders. In addition, some differences were identified between Libyan men and women in terms of unhealthy eating habits and obesity such as gender disparities in the consumption of SSBs. The findings of the study reveal areas for action for Libyan researchers, clinicians, policymakers, and government officials about UEHs in the Libyan context. This could subsequently inform establishing and developing new interventions for preventing and controlling the obesity epidemic through food system improvements. The Libyan authorities should improve the availability of affordable healthier foods and beverages in public-service venues. Promoting high nutrient-dense foods choices could be achieved through instead of heavily subsiding staple food commodities that fuel obesity, the Libyan government should reduce these subsidies and subsidize high nutrient-dense foods instead, particularly in deprived areas. While the study has identified many unhealthy eating behaviors that might contribute to fuel the obesity epidemic in Libyan adults of both genders, a number of other modifiable risk factors need to be studied such as physical activity, sedentary behaviors and the obesogenic environment. Due to the unstable political situation in Libya, it is critical to examine how political instability in Libya may affect everyday behaviors such as discouraging residents to access high nutrient-dense foods. Furthermore, more innovative and rigorous research is needed to draw causal inferences, such as longitudinal studies and nationally-representative surveys to describe secular trends in obesity. Finally, purely qualitative approaches are also urgently needed to elicit people’s beliefs, experiences and perceptions in order to continue developing our understanding of unhealthy eating behaviors as potential factors leading to the obesity epidemic.

## Figures and Tables

**Table 1:** Grouping and Recoding Variables.

Variable	Variable Recoding into Two Categories for Binary Regression Analysis
Marital Status	Single: Never married. Married: Married, divorced, separated, and widowed.
Level of education	Low Level: No formal schooling, less than primary school and primary school completed Moderate level: Secondary school and high school completed. High level: College/university completed. Post-graduate degree.
Occupation	Employed: Government employee, non-government employee, self-employed Unemployed: retired, housework, unemployed (able to work) and unemployed
Income (Libyan Dinar = ½ Pound)	Low income: <500 and 500–999. Moderate income: 100–1999 and 2000–2999. High income: 3000–3999 and ≥4000
Fast-food consumption	Infrequent fast-food consumption in a day: ≤1 time. Excessive consumption of fast food in a day: ≥2 times. Infrequent fast-food consumption in a week: ≤4 times. Excessive consumption of fast food in a week: ≥5 times.
SSBs consumption	Infrequent SSBs consumption in a day: ≤1 time. Excessive SSBs consumption in a day: ≥2 times. Infrequent SSBs consumption in a week: ≤6 times. Excessive SSBs consumption in a week: >6 times.
Fruits and vegetable Consumption	Inadequate fruits and vegetables intake in a day: ≤3 times. Adequate fruits and vegetable intake in a day: >3 times. Inadequate fruits and vegetables intake in a week: ≤3 times. Adequate fruits and vegetable intake in a week: >3 times.
Skipping breakfast	A high frequency of skipping breakfast every day on a daily basis A high frequency of skipping breakfast in a week: >3 times.
Consumption large food portion sizes at one sitting	Never and rarely large FPS consumed at one sitting Occasionally and always large FPS consumed at one sitting
The dichotomous outcome of obesity (BMI)	Not obese (BMI < 30 kg/m^2^) Obese (BMI ≥ 30 kg/m^2^)

**Table 2:** Demographic and anthropometric measurement characteristics of the participants (*n* = 401).

Demographic and Socio-Economic Characteristics	Female (F) *n* (%)	Male (M) *n* (%)	Total *n* (%)
Gender	253 (63)	148 (37)	401 (100)
Age			
20–29	51 (20)	27 (18)	78 (19)
30–39	56 (22)	27 (18)	83 (21)
40–49	78 (31)	37 (25)	115 (29)
50–59	32 (13)	18 (12)	50 (12)
60–65	36 (14)	39 (26)	75 (19)
Marital Status			
Single	85 (33.6)	47 (31.8)	132 (32.9)
Married	152 (60)	88 (59.5)	240 (59.9)
Divorced	10 (4)	6 (4.1)	16 (4)
Widowed	5 (2)	2 (1.4)	7 (1.7)
Separated	1 (0.4)	5 (3.4)	6 (1.5)
Racial group			
Arabic	210 (83)	129 (87.2)	339 (84.6)
Berbers ‘Imazighen’	28 (11.1)	15 (10.1)	43 (10.7)
Toubou	15 (5.9)	4 (2.7)	19 (4.7)
Level of education:			
No formal schooling	17 (6.7)	13 (8.8)	30 (7.5)
Less than primary school	16 (6.3)	14 (9.5)	30 (7.5)
Primary school completed	12 (4.7)	5 (3.4)	17 (4.2)
Secondary school completed	45 (17.8)	20 (13.5)	65 (16.2)
High school completed	32 (12.6)	21 (14.2)	53 (13.2)
College/university completed	98 (38.7)	57 (38.5)	155 (38.7)
Post graduate degree	33 (13)	18 (12.2)	51 (12.7)
Occupation			
Government employee	116 (45.8)	64 (43.2)	180 (44.9)
Non-government employee	22 (8.7)	24 (16.2)	46 (11.5)
Self-employed	32 (12.6)	15 (10.1)	47 (11.7)
Non-paid	20 (7.9)	16 (10.8)	36 (9)
Student	1 (0.4)	1 (0.7)	2 (0.5)
Housework	27 (10.7)	10 (6.8)	37 (9.2)
Retired	2 (.8)	1 (0.7)	3 (0.7)
Unemployed (able to work)	27 (10.7)	17 (11.5)	44 (11)
Unemployed (unable to work)	6 (2.4)	0 (0)	6 (1.5)
Monthly Income: “(LYD)” *			
<500	6 (2.4)	5 (3.4)	11 (2.7)
500–999	48 (19)	38 (25.7)	86 (21.4)
1000–1999	19 (7.5)	11 (7.4)	30 (7.5)
2000–2999	56 (22.1)	17 (11.5)	73 (18.2)
3000–3999	100 (39.5)	59 (39.9)	159 (39.7)
≥4000	24 (9.5)	18 (12)	42 (10.5)
Anthropometric measurements	Female Mean (±SD)	Male Mean (±SD)	Participants Mean (±SD)
BMI values (kg/m^2^)	30.12 (±6.54)	28.50 (±5.40)	29.52 (±6.19)
Visceral Fat Rating (1–12)	10.65 (±4.2)	10.04 (±3.9)	10.42 (±4.1)
Body fat %	34.24 (±9.51)	27.01 (±7.31)	31.57 (±9.42)
Three weight status categories (BMI categories)	Female N (%)	Male N (%)	Prevalence of overweight and obesity Mean (±SD)
Normal weight (BMI = 18.5–24.9 kg/m^2^)	50 (33.8)	49 (19.4)	99 (24.7)
Overweight (BMI = 25–29.9 kg/m^2^)	48 (32.4)	84 (33.2)	132 (32.9)
Obese (BMI ≥ 30 kg/m^2^)	50 (33.8)	120 (47.4)	170 (42.4)

(One Libyan Dinar (LYD) * = 0.50 GBP [4] (Currently exchange rate LYD = 0.16 GBP).

**Table 3:** Characteristics of unhealthy eating habits among Libyan adults.

Unhealthy Eating Habits	Mean (±SD) TIMES/a Typical Day			Mean (±SD) Times/a Typical Week			Spearman’s Rank Test Value (rho)	*p*-Value
	Male	Female	Total	Male	Female	Total		
The amount of fast food intake	1.23 (±0.7)	1.46 (±0.8)	1.37 (±0.8)	5.51 (±2.9)	5.62 (±3.1)	5.54 (±3.1)	0.440	0.000 **
The amount of sugar sweetened beverages intake	3.0 (±.25)	3.5 (±0.5)	3.15 (±1.2)	10.18 (±1.5)	11.55 (±1.35)	11.68 (±1.23)	0.467	0.000 **
The amount of vegetable and fruit intake	2.47 (±1.3)	2.57 (±1.1)	2.53 (±1.2)	10.33 (±1.4)	9.17 (±1.9)	10.1 (±1.6)	0.081	0.107
The number of times skipping breakfast	0.5 (±0.6)	0.6 (±0.6)	0.5 (±0.3)	3.33 (±1.9)	3.9 (±1.7)	3.18 (±1.83)	−0.506	0.000 **
Consumption of a full large portion size meal served at one sitting	Male (M) N (%)		Female (F) N (%)		Total N (%)		Chi-square (X^2^)	*p*-Value
Always	71 (48)		95 (37.5)		166 (41.4)		13.8	0.032 *
Occasionally	26 (17.6)		47 (18.6)		73 (18.2)			
Rarely	21 (14.2)		46 (18.2)		67 (16.7)			
Never	30 (20.3)		65 (25.7)		95 (23.7)			

* *p* < 0.05, ** *p* < 0.01.

**Table 4:** The impact of multiple independent variables (unhealthy eating habits) on obesity among Libyan adults of both genders using binary logistic regression.

Variables for Unhealthy Eating Habits	Sig.	Exp(B)	95% CI for EXP(B)	
			Lower	Upper
1. The amount of fast-food intake in a typical day.	0.042 *	3.14	1.04	9.46
2. The amount of fast-food intake in a typical week.	0.000 **	6.83	2.59	12.01
3. The amount of sugar-sweetened beverages intake in a typical day.	0.316	.61	0.23	1.61
4. The amount of sugar-sweetened beverages intake in a typical week.	0.051	2.66	0.99	7.10
5. The amount of vegetable and fruit intake in a typical day.	0.510	1.42	0.50	4.05
6. The amount of vegetable and fruit intake in a typical week.	0.339	0.63	0.240	1.63
7. The number of times skipping breakfast in a typical day.	0.03 *	0.67	0.03	0.25
8. The number of times skipping breakfast in a typical week.	0.000 **	0.08	0.45	0.67
9. Consumption of a full large portion size meal, served at one sitting.	0.000 **	4.78	1.84	12.46

* *p* < 0.05, ** *p* < 0.01.

**Table 5:** Association between unhealthy eating habits and BMI using binary logistic regression across gender-related variances when sociodemographic and SES factors were adjusted ^1^.

Variables for Unhealthy Eating Habits	Male				Female				
	Sig *p*-Value	Odds Ratio (OR)	95% CI for Exp(B)		Sig *p*-Value	Odds Ratio (OR)	95% CI for Exp(B)		
			Lower	Upper			Lower	Upper	
1. Age	0.05 *	0.015	0.000	1.07	0.01 *	11.7	1.65	83.02	
2. Marital Status	0.91	0.86	0.07	10.40	0.37	0.54	0.13	2.11	
3. Ethnicity	0.82	1.91	0.01	447.40	0.50	2.45	0.18	32.56	
4. Education level	0.04 *	0.03	0.001	0.79	0.001 **	0.05	0.01	0.30	
5. Occupation	0.03 *	14.1	1.53	35.50	0.19	2.73	0.61	12.17	
6. Income level	0.416	1.68	0.48	5.92	0.59	1.22	0.59	2.55	
7. The amount of fast-food intake in a typical day.	0.047 *	2.52	4.04	12.32	0.043	2.14	3.32	11.12	
8. The amount of fast-food intake in a typical week.	0.014 *	4.65	1.04	9.46	0.002 **	5.50	1.88	16.11	
9. The amount of sugar-sweetened beverages intakes in a typical day.	0.094	0.089	0.01	1.52	0.810	0.87	0.298	2.61	
10. The amount of sugar-sweetened beverages intake in a typical week.	0.452	2.53	0.22	28.61	0.013 *	4.02	1.35	11.99	
11. The amount of vegetable and fruit intake in a typical day.	0.408	2.86	0.24	34.50	0.580	0.72	0.23	2.30	
12. The amount of vegetable and fruit intake in a typical week.	0.379	0.34	0.03	3.83	0.538	0.72	0.25	2.08	
13. The number of times breakfast intake in a typical day.	0.042 *	0.02	0.01	0.77	0.000 **	0.11	0.03	0.43	
14. The number of times breakfast intake in a typical week.	0.011 *	0.03	0.01	0.24	0.005 **	0.21	0.08	0.63	
15. Full portion size of served food consumed at one sitting	0.027 *	19.5	1.41	27.74	0.024 *	3.40	1.18	9.84	

OR: odds ratio; CI: confidence interval. The OR was calculated for all variables with 95% confidence intervals. ^1^ Adjusted for, gender, age, marital status, level of education, occupation, and income. * *p* < 0.05, ** *p* < 0.01.

## Data Availability

The data in this paper is largely from a thesis submitted by Hamdi Abdulla A. Lemamsha in partial fulfilment of the requirements or the Degree Doctor of Philosophy in adult obesity: causes and consequences, prevention and management, University of Bedfordshire, 2016. No new data were created or analysed in this study. Data sharing is not applicable to this article.

## Abbreviations

BMI	Body Mass Index
SES	socioeconomic status
SSBs	Sugar-Sweetened Beverages
FV	Fruit and vegetable
FPS	Food Portion Size
SEM	Socio-Ecological Model
SBF	Skipping Breakfast
FFR	Fast Food Restaurant
